# Touch localization after nerve repair in the hand: insights from a new measurement tool

**DOI:** 10.1152/jn.00271.2023

**Published:** 2023-09-20

**Authors:** Martin Weber, Andrew Marshall, Ronan Timircan, Francis McGlone, Simon J. Watt, Obi Onyekwelu, Louise Booth, Edwin Jesudason, Vivien Lees, Kenneth F. Valyear

**Affiliations:** ^1^School of Psychology and Sport Sciences, Bangor University, Bangor, United Kingdom; ^2^Department of Musculoskeletal Biology, Institute of Life Course and Medical Sciences, University of Liverpool, Liverpool, United Kingdom; ^3^Institute of Psychology, Health and Society, University of Liverpool, Liverpool, United Kingdom; ^4^Department of Plastic Surgery, Portsmouth Hospitals University NHS Trust, Cosham, United Kingdom; ^5^Department of Orthopaedics and Trauma, Betsi Cadwaladr University Health Board, Bangor, United Kingdom; ^6^Department of Plastic Surgery, University of Manchester, Manchester, United Kingdom; ^7^Manchester University Foundation Hospitals Trust, Manchester, United Kingdom

**Keywords:** locognosia, nerve injury, nerve repair, reinnervation errors, touch localization

## Abstract

Errors of touch localization after hand nerve injuries are common, and their measurement is important for evaluating functional recovery. Available empirical accounts have significant methodological limitations, however, and a quantitatively rigorous and detailed description of touch localization in nerve injury is lacking. Here, we develop a new method of measuring touch localization and evaluate its value for use in nerve injury. Eighteen patients with transection injuries to the median/ulnar nerves and 33 healthy controls were examined. The hand was blocked from the participant’s view and points were marked on the volar surface using an ultraviolet (UV) pen. These points served as targets for touch stimulation. Two photographs were taken, one with and one without UV lighting, rendering targets seen and unseen, respectively. The experimenter used the photograph with visible targets to register their locations, and participants reported the felt position of each stimulation on the photograph with unseen targets. The error of localization and its directional components were measured, separate from misreferrals—errors made across digits, or from a digit to the palm. Nerve injury was found to significantly increase the error of localization. These effects were specific to the territory of the repaired nerve and showed considerable variability at the individual level, with some patients showing no evidence of impairment. A few patients also made abnormally high numbers of misreferrals, and the pattern of misreferrals in patients differed from that observed in healthy controls.

**NEW & NOTEWORTHY** We provide a more rigorous and comprehensive account of touch localization in nerve injury than previously available. Our results show that touch localization is significantly impaired following median/ulnar nerve transection injuries and that these impairments are specific to the territory of the repaired nerve(s), vary considerably between patients, and can involve frequent errors spanning between digits.

## Graphical abstract

**Figure:**
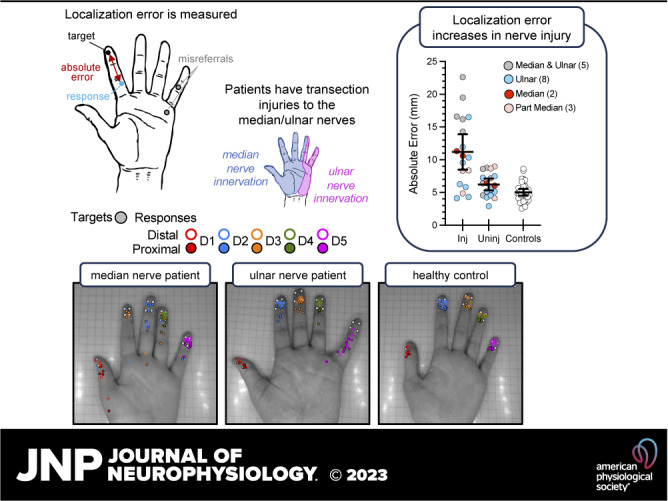

## INTRODUCTION

Injuries to the nerves of the hand are common and have significant longstanding consequences. When a nerve is cut in adulthood, complete recovery is not expected. Sensory and motor impairments, and often pain, persist indefinitely (1–3). A major challenge, thought to limit recovery, is that nerve regeneration following surgical repair is not topographically guided (4–7). Sprouting fibers establish new connections, innervating end receptors at different locations relative to the preinjury organization. These rewiring events, known as targeting or reinnervation errors, are difficult to measure directly in humans; yet one accepted proxy is the presence and character of aberrant touch localization. In this study, we develop an improved method for measuring touch localization on the hand and evaluate its value for use in nerve injury.

The classic literature on peripheral nerve injury is richly populated with accounts of aberrant touch localization. At the turn of the 20th century, several independent investigators voluntarily had their own nerves cut and sutured for the purpose of experimentation (8–10). Self-observation featured both introspective and objective measures, and aberrant touch localization was extensively reported. Early clinical observations of aberrant touch localization were also extensively documented (11, 12), and in a cogent report featuring selected patient cases, Hawkins (13) highlights the clinical significance of aberrant touch localization as a positive marker of nerve regeneration success after nerve repairs. It was said that “[aberrant localisation] can always be elicited when sensory regeneration of a sutured nerve has occurred.” Without quantitative group results based on rigorous statistical methods, however, what can be understood from the classic literature is limited.

An elegant method for quantifying touch localization on the hand was introduced by Noordenbos (14) and further developed by his student Hamburger (15). In this approach, which we call the “red-lens method,” participants wear a set of glasses with red lenses. While blocked from the participant’s view, the experimenter uses a pen to mark the hand. The marks serve as targets for touch stimulation, visible to the experimenter yet invisible to the participant when viewed through the red lenses. Following stimulation of a given target, the participant reports where they felt they were touched using a different colored pen, making their own mark on the hand. Measurement of the distance between stimulated and felt locations can be taken, directly, in continuous units. This measurement is known as the error of localization.

Hamburger used this method to characterize touch localization in healthy controls, revealing, for example, a distal-proximal gradient in the error of localization, with the distal fingertips outperforming the middle and proximal pads of the digits and the palm (15). Original clinical applications were limited, however. Four patient case studies were provided—two with hand-nerve injuries and two with brain injuries—and the results were purely descriptive. No quantitative comparisons were made.

The first quantitative applications of the red-lens method to clinical populations were made by Braune and Schady (16). Eleven patients with complete median/ulnar nerve transection injuries were tested. The findings revealed increased error of localization for responses within the territory of the injured nerve compared with homologous locations on the uninjured hand. Surprisingly, impairments were restricted to the middle and proximal digit pads; touch localization at the distal digit pads was no different between injured and uninjured sides. This result conflicts with expectations based on peripheral regrowth, where reinnervation takes longer to complete at more distal sites from the repair, and was attributed to central factors.

A variation of the red-lens method was used to measure touch localization after major hand reconstruction (17). Three patients who had undergone complete hand replantation and two hand transplant recipients were tested. Error of localization was increased for the repaired hand, and longitudinal tests (taken only in the transplant patients) showed marked improvements over time. Conclusions from this study are limited, however, as statistical comparisons are based on a small sample of patients.

The red-lens method has significant limitations. First, it is difficult to take repeated measures from the same targets. Each new measurement requires clearing the marks from prior responses. Accordingly, much of the original results of Hamburger (15) are based on single measurements. This makes it necessary to average across targets for statistical analyses, limiting spatial resolution. Second, it can be difficult to acquire accurate and precise measurements without the experimenter touching the participant’s skin. Contact with the skin will provide additional cues, and, thus, is to be avoided. Yet, to do so, the measurement instrument (e.g., calliper) must be held away from the skin surface, limiting accuracy and precision, and, possibly, measurement consistency. Otherwise, if measurements are performed after testing is complete, tracking which responses belong to which targets is challenging. With numerous targets and/or multiple responses per target, this would be difficult to achieve. Finally, although not necessarily a limitation, participants touch their skin to record responses. This provides an opportunity to compare felt stimulation against felt (and viewed) responses, which may improve future performance. The method we developed in the current study addresses these limitations.

Other research has used an “area of localization” method to evaluate touch localization in hand-nerve injury. In the modified Marsh method developed by Christina Jerosch-Herold (18, 20–22; see also, original work by Marsh, 1990, 19), the distal pads of the digits are divided into quadrants, comprising 20 zones. Touch is applied to each zone, and the participant verbally reports which zone they felt was touched (while viewing a diagram of a hand with the zones labeled). Performance is measured as a score: 2 points for the correct zone; 1 point for an adjacent zone; 1 point for the homologous zone of a neighboring digit; 0 points for other responses. Applied to patients with hand-nerve injuries, the modified Marsh method shows high sensitivity to impairment, high external validity, and excellent test-retest and interrater reliability (18, 20–23). The test is standardized and simple to administer, and the materials are affordable and easily portable, test properties that are of high value for clinical research and assessment.

The modified Marsh method also has significant limitations, however. The error of localization is not measured. Performance scores reflect arbitrary units averaged across zones, and spatial resolution is ultimately limited by zone size. Errors that span between digits, which we call misreferrals (see methods, *Dependent errors*), are scored but not otherwise distinguished. To better understand the nature of localization deficits in nerve injury, such as their potential relationship with reinnervation errors, we believe it will be necessary to capture more detailed features, including absolute and directional errors of localization and misreferrals. The method developed in the current study enables rigorous quantification of these features.

The purpose of this study was to develop an improved method of measuring touch localization and evaluate its value for use in nerve injury. Addressing the significant methodological limitations described earlier, our method enables detailed quantification of the error of localization, multiple measurements from the same skin locations in a repeatable and efficient manner, and eliminates the need to measure error directly from the participant’s hand. Also, participant responses do not involve touching the hand, making our assessment of touch localization unconfounded by the possible influence of response feedback.

We use our new method to evaluate touch localization in 18 individuals with transection injuries to either the ulnar or median nerves, or both. Thirty-three healthy controls are tested for comparison. The method generates a rich profile of information at the level of individual participants and across different parts of the hand. Our findings provide a more comprehensive evaluation of touch localization in nerve injury than previously available, revealing significant increases in the error of localization within the projection territory of the repaired nerve(s).

## METHODS

### Participants

#### Patients.

Eighteen patients completed testing (age range: 21–75 yr; mean age: 38.3 yr; 7 females). Most patients had complete transection injuries: eight ulnar, two median, and five ulnar and median (“both”). The remaining three patients had incomplete transection injuries of the median nerve. All patients underwent surgical repairs within 24 h of injury. One patient’s injury, a partial median transection, was due to self-harm. This patient was deemed mentally stable when tested. All other injuries were of traumatic origin.

All patients had sustained their injuries in adulthood (mean: 34.8 yr; median: 33 yr; range: 17–68 yr). Time-since-repair ranged from 8 to 130 mo (mean: 42.3 mo; median: 34 mo). Two patients were left-handed according to the modified version of the Waterloo Handedness Inventory (24; scores range from −30 to +30). Seven patients had injured their dominant hand. See Table 1 for complete demographic details.

**Table 1.** Patient demographics and standardized test scores

	Demographics						Standardized Tests				
										Marsh	
Subject	Sex	WS	Age	MSR	Side	Nerve	DASH	McGill	Rosen	Inj.	Uninj.
*P1*	F	28	29	19	L	M	29	15	0.15		
*P2*	M	30	32	10	L	U	21	3	0.23		
*P3*	M	30	51	60	R	U	66	14	0.51	63	92
*P4*	F	30	26	37	R	M + U	53	13	0.13	96	63
*P5*	F	−8	23	62	R	U	4	1	0.86	100	96
*P6*	F	30	39	26	L	U	10	0	0.58	100	96
*P7*	M	15	68	75	R	U	33	20	0.86	71	92
*P8*	M	30	41	82	R	M + U	22	14	0.28		
*P9*	F	29	37	11	L	M	18	1	0.26	73	68
*P10*	F	30	24	8	L	M + U	28	17	0.00		
*P11*	M	30	31	28	L	M + U	26	15	0.18		
*P12*	M	30	41	18	R	M + U	15	4	0.14	65	98
*P13*	M	30	45	30	L	M (part.)	21	36	0.60	70	75
*P14*	M	22	34	31	R	M (part.)	3	1	0.94	96	89
*P15*	F	27	21	45	R	U	8	2	0.23	83	100
*P16*	M	30	39	49	L	M (part.)	0	0	0.91	91	88
*P17*	M	−30	75	130	R	U	9	3	0.25	50	83
*P18*	M	3	34	40	L	U	23	5	0.29	92	88

The DASH score reflects the level of difficulty experienced using the upper limb to perform activities of daily living. A score of 0 indicates no difficulty; a score of 100 indicates maximal difficulty. The (short-form) McGill score reflects severity of pain experienced. A score of 0 indicates no pain; a score of 45 indicates maximal pain.

DASH, Disabilities of the Arm, Shoulder, and Hand; F, female; inj., injured hand; L, left; M, male; M, median; MSR, months since repair; part., partial injury; R, right; U, ulnar; uninj., uninjured hand; WS, Waterloo score (−30 to 30; negative values = left handedness, positive values = right handedness).

#### Healthy controls.

Thirty-three control participants completed testing (age range: 19–63 yr; mean age: 31.9 yr; 13 females). Two participants were left-handed.

All participants gave written informed consent before taking part in the study. Procedures were approved by the Bangor University School of Human and Behavioural Sciences Ethics Board. Patients and 28 healthy controls completed the reported tests as part of a larger study, also involving functional MRI. These data will be reported elsewhere. The tests reported here took ∼90 min to complete. Participants received financial compensation.

### Locognosia: Digital Photograph Method

#### Setup and materials.

**Figure 1.**
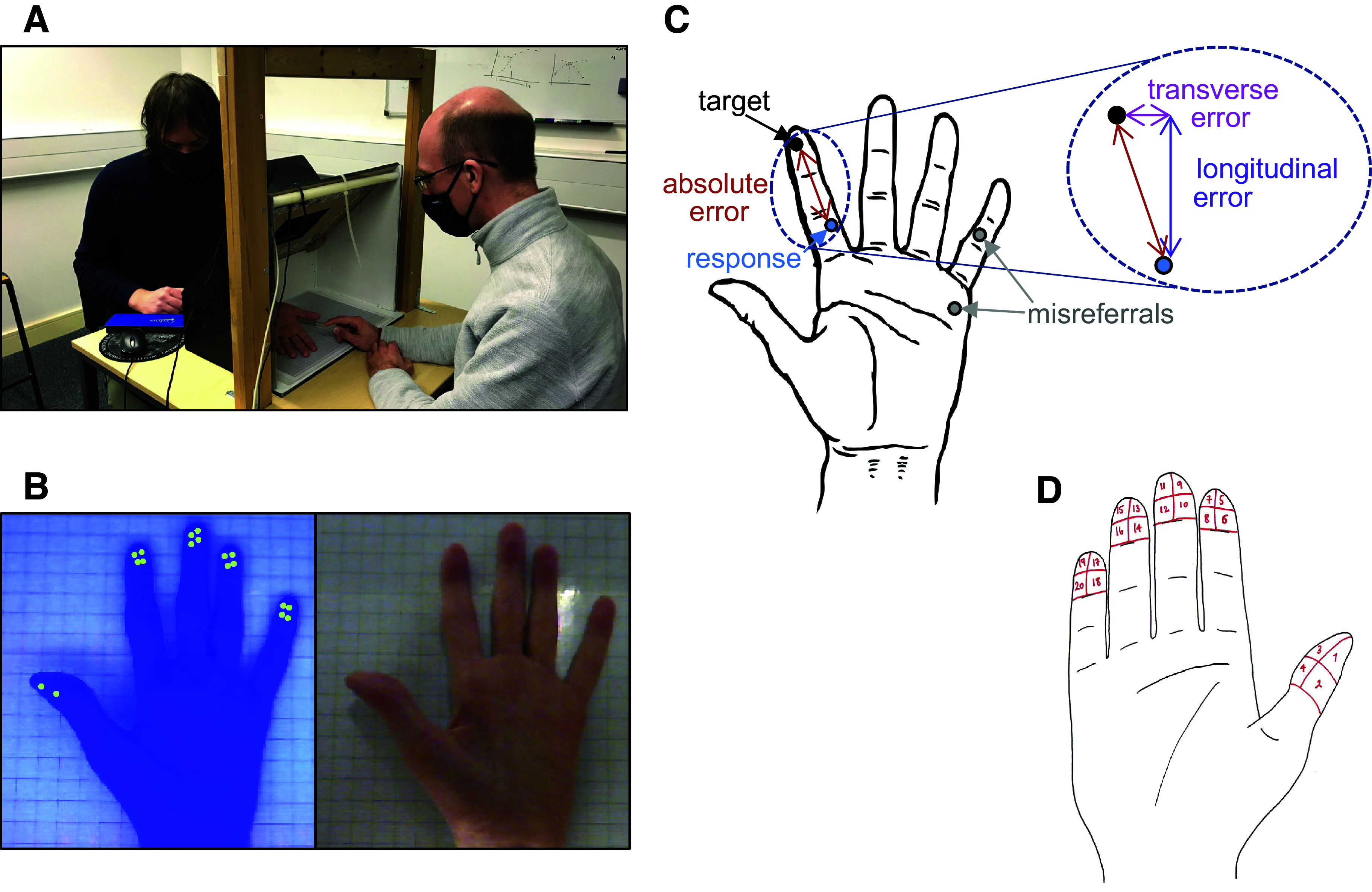
Methods. *A*: position of experimenter (right) and participant (left) during the digital photograph method. The blinder box can be seen in the center. *B*: UV-light image (*left*) that was used by the experimenter to register the targets and a normal light image (*right*) on which the participant registered their response. *C*: target is the location where the experimenter applies the touch stimulus. Response is where the participant indicates where they felt they were touched. The absolute localization error is computed as the Euclidian distance between target and response. The absolute error can be decomposed into a longitudinal component (along the axis of the finger) and a transverse component (perpendicular to the axis of the finger). Note, the *inset* does not accurately depict longitudinal/transverse components, but is shown for conceptual visualization purposes only. Responses made to another digit or to the palm are defined as misreferrals. *D*: right-hand diagram used for the modified Marsh method (image taken with permission from C. Jerosch-Herold, University of East Anglia; link to resources: https://www.uea.ac.uk/about/school-of-health-sciences/research/resources-and-tools/the-locognosia-test). A corresponding left-hand diagram was provided when the left hand was tested. UV, ultraviolet.

Participants were seated at a table in a well-lit room. On the table, there was a wooden blinder box with a small hole, for which the participants put their arm through with the palm of the hand facing up (Fig. 1*A*). Wrist and hand cushions were provided for comfort. The box was open on the other side to allow the experimenter to deliver the stimuli. A Logitech C270 webcam and switchable ultraviolet (UV) lights were mounted to the ceiling of the box. A monitor and mouse were placed at the side of the participant’s body that was currently not being tested—the “participant monitor.” The monitor displayed a picture of the participant’s hand, and the mouse was used to register responses (see *Procedure*). A second monitor, the “experimenter monitor,” faced the experimenter and was positioned so that participants could not see what was displayed.

Stimuli were delivered manually by the experimenter using a 6.1 Semmes-Weinstein monofilament with a peak force of 100 gf. This level of force was suprathreshold for 17/18 patients and for all healthy controls. The remaining patient reported difficulty feeling the 6.1 filament and was tested using the 6.65 Semmes-Weinstein monofilament with a peak force of 360 gf. This patient had a median nerve injury, tested at 19 mo since repair.

A custom-written Visual Basic program was used to control the experiment.

#### Procedure.

The participant placed their hand through the blinder box. The experimenter marked 18 points on the volar surface of the participant’s hand using a UV pen. Four points were made on the distal pad of each finger in an arrangement divided into relative ulnar/radial-distal/proximal positions (Fig. 1*B*), matching the target “zones” of the modified Marsh method (Fig. 1*D*; see *Modified Marsh method*). Two points were marked on the radial side of the distal pad of the thumb. The ulnar side of the thumb was untested due to technical challenges. This differs from the modified Marsh method and represents a limitation of the digital photograph method.

After the targets were marked, the participant was asked to open and flatten their hand against the base of the apparatus while two photographs were taken in succession (1 s apart), one with and one without UV lighting (Fig. 1*B*). It is important to appreciate that during testing participants assumed a relaxed posture, described below. Flattening the hand was only required for the brief few seconds for which the photographs were taken. The experimenter used the photograph with UV lighting to register the x- and y-coordinates of each target. This was done using a mouse to manually indicate the center of each UV mark. The photograph with UV lighting was displayed on the “experimenter monitor,” visible only to the experimenter.

After target registration was complete, the photograph of the hand with normal lighting (and targets invisible) was displayed to the participant. This image was oriented such that participants would see their hand as it was positioned within the blinder box from their own perspective, with the fingertips pointing upward, and was made visible to participants throughout the experiment. Each target was then stimulated in turn by the experimenter. To know which target to stimulate on a trial-by-trial basis, a normal-light image of the participant’s hand was displayed on the experimenter monitor indicating the location of a target with a dot and its corresponding target number. This image was oriented to align with the experimenter’s view of the participant’s real hand, with the fingers pointing downward.

Stimulation was delivered for approximately 1 s and accompanied by a verbal cue “now” from the experimenter. Participants then used a computer mouse to indicate the felt position of each stimulation on the photograph of their hand with normal lighting. After each response was indicated, a pop-up window appeared in the center of the screen that asked participants to confirm their choice. This allowed participants to correct their choice in case they accidentally missed the location they wanted to click. Confirmed responses registered the x- and y-coordinates of the cursor. Participants were instructed to keep their hand still during stimulation and to avoid moving between stimulation and choosing their response. Moving the fingers after stimulation can improve localization performance (15). Participants were asked to find a comfortable posture, with the hand open yet relaxed. Supporting cushions were provided, as needed. It was not necessary to flatten the hand against the base of the apparatus during testing, as for the photograph. Sometimes participants had to be asked to reopen the hand during testing, if the fingers were curled inward such that access to targets with the monofilament was difficult. No feedback regarding performance was given to the participant during the experiment.

Before collecting any responses, the experimenter applied stimulation to two or three different targets so that the participant could experience how a trial felt. After these initial trials, the testing began. The complete test comprised five blocks of 18 trials, per hand. In each block, all 18 possible target locations were stimulated, and target order was randomized within blocks. Participants were not told that all targets would be stimulated once per block. Rest breaks were permitted throughout testing and encouraged between blocks. Some participants found it fatiguing to keep their hand in an appropriate posture, and thus took more breaks. Participants were allowed to take their hand out of the blinder box during breaks, but were asked not to study their hand closely. For each hand, the test took ∼20 min to complete.

Both hands were tested. For patients, the injured hand was always tested first. This was done to prioritize measurement of the injured hand, in the event that a patient decided to discontinue testing. This did not occur. For healthy controls, hand order was counterbalanced between participants. Among the other tests reported here, see below, the digital-photograph method was always completed first (aside from the control task, see next).

#### Control task.

A short control task was implemented to begin the experiment. This control task involved participants using a computer mouse to indicate the position of 12 visible dots on a photograph of a lettered six-by-two grid. The dots were labeled with letters A-through-H. The experimenter asked the participant to click the dot corresponding to each letter in succession, presented in a random order. The setup was identical to the main task, using the same monitor and mouse positioning. This control task was done to evaluate whether participants had difficulties controlling the mouse. The task was done for each hand. The error of localization (as calculated below) was negligible for all participants. Performance on the main task could therefore not be attributable to movement difficulties.

#### Dependent measures.

*Absolute error.* The differences between x- and y-coordinates of each target-response pair were first computed in pixels, and then converted to millimeters. The conversion from pixels to millimeters was done using conversion factors defined separately for x- and y-dimensions. The conversion factors were derived by measuring known distances in the picture, based on the background grid, using ImageJ version 1.53k. Specifically, the experimenter used a mouse to manually indicate points in the grid, separately for x- and y-dimensions, and the corresponding pixels-to-mm conversions were computed. Absolute error was then calculated as the Euclidian distance between target-response pairs using x- and y-error in millimeters.

$$E_{absolute}=\sqrt{E_{x}^{2}+E_{y}^{2}}$$

Absolute error is otherwise known as the error of localization.

*Directional error.* We were also interested in examining directional error, preserving the constituent directionality of the error of localization. This way, we can evaluate evidence of bias—systematic directionality in responses to stimulation of a given digit. The longitudinal error is defined in the proximal-distal axis along the length of a digit, and the transverse error is defined in the ulnar-radial axis along the width of a digit. To calculate each, an angle per digit was defined in the photograph of each hand, using ImageJ. The angle was measured with reference to the lower edge of the image and an extended line was drawn by the experimenter through the midline of the digit being measured. The longitudinal and transverse errors were then calculated as follows:

$$E_{longitudinal}=E_{x}\times \text{sin}\left(\theta_{digit}\right)+E_{y}\times \text{cos}\left(\theta_{digit}\right)$$

$$E_{transverse}=E_{x}\times \text{cos}\left(\theta_{digit}\right)+E_{y}\times \text{sin}\left(\theta_{digit}\right)$$

*Misreferrals.* Trials where responses were made to an incorrect digit, or to the palm of the hand were defined as misreferrals. To identify misreferrals, responses were displayed on the image of the hand, and color coded according to which digit had been stimulated (using R). The experimenter then visually identified misreferrals as those responses that were made on an incorrect digit, or the palm. Responses made over the permanent crease separating digits and the palm were counted as belonging to the corresponding digit. Those below the crease were defined as misreferrals to the palm.

Absolute and directional errors, described above, were computed excluding misreferrals. This was done since interpretation of error in the case of misreferrals is problematic. In the case of misreferrals, it is unclear how error should be defined, using a straight line between targets and responses, or the shortest path along the skin’s surface, for example. Furthermore, the magnitude of the error in the case of digit-to-digit misreferrals would depend on relative digit position—i.e., whether the digits were together or apart. As such, misreferrals were analyzed separately.

#### Analyses.

*Absolute error.* To evaluate impairment, target locations on the injured hand of patients were defined as either within or outside the territory of the injured nerve. Targets within the territory of the injured nerve comprised the “Inj” condition. For isolated median nerve injuries, Inj targets comprised all locations on digits D1-to-D4 (14 targets). For isolated ulnar nerve injuries, Inj targets comprised all locations on digits D4-to-D5 (8 targets). For patients with injuries to both median and ulnar nerves, all targets on the injured hand were defined as Inj targets.

Canonically, the division between median and ulnar nerve territories runs through the middle of D4, so radial targets on D4 are within the median nerve territory while ulnar targets are within the ulnar nerve territory. There is overlap through communicating branches, however, and the precise anatomy varies between individuals (25–27). Thus, we defined all D4 targets as within the Inj condition for both median and ulnar nerve patients, as noted above. If anything, this may underestimate impairment, since we may be including within the Inj condition target locations that are serviced by an intact median/ulnar nerve, accordingly.

For comparison, the homologous target locations on the uninjured hand were defined as the “Uninj” condition. All statistical comparisons between Inj and Uninj conditions were made using paired *t* tests. In the case of violations of normality, the Wilcoxon matched-pairs signed-rank test was used. Normality was tested using the Shapiro–Wilk test.

As the patient group comprised a mixture of isolated median, isolated ulnar, and both ulnar and median nerve injuries, we needed to organize our data from healthy controls so that fair comparisons between patients and controls could be made. Otherwise, group comparisons would involve estimates of error from different combinations of digits. As such, the controls’ data were formulated to “match” the patient group based on the proportions of patients with median/ulnar/both nerve injuries. Specifically, five patients had isolated median nerve injuries (∼28% of the group); thus, nine controls were treated as median nerve-injured (∼27% of the control group). This meant that the data from these nine controls comprised responses from all targets on digits D1-to-D4 (14 targets), matching the median nerve patients. In the same fashion, eight patients had isolated ulnar nerve injuries (∼44%), and so, 15 controls were assigned to match these patients (∼45% of the control group). Their data comprised all targets on D4 and D5 (eight targets), matching the ulnar patients. The remaining proportion of controls was matched against the patients with both median and ulnar nerve injuries. Their data were taken from all targets. The assignment of controls to patient subgroups was otherwise random. Once this matched control group was defined, a paired-sample *t* test was used to evaluate whether there were differences between the dominant and nondominant hands. If no differences were observed, the controls’ data were averaged across hands.

All statistical comparisons of the absolute error of localization between patients and controls were made using unpaired *t* tests. If the variances between groups were unbalanced, a Welch’s correction was applied. If the residuals of the initial tests were not normally distributed, as measured using Shapiro–Wilk, nonparametric Mann–Whitney tests were used.

*Directional error.* To evaluate whether the longitudinal or transverse components of the error of localization showed any systematic biases, one-sample *t* tests were performed (against zero). This was done separately for *digits 2* and *5*, and per patient and control groups, as part of our digit-specific analyses. The critical *P* values were corrected for multiple comparisons using Bonferroni corrections.

*Misreferrals.* Misreferrals were identified as described above. Although typically few misreferrals per individual were observed, and for some participants, no misreferrals were made, we nonetheless carried out the following analyses.

First, we wanted to evaluate whether patients showed a greater number of misreferrals due to their nerve injury. To do so, we converted the total number of misreferrals observed per hand to proportions, dividing by the total number of trials (i.e., 90 per hand). For subsequent statistical comparisons, we arcsine transformed these data, calculated as the arcsine square root of the proportions. This makes the resultant distributions more symmetrical and reduces problems with violations of the assumption of normality. This is appropriate to do when numerous scores are near ceiling/floor.

We then compared arcsine-transformed proportions of misreferrals for the injured hand of patients against that of controls. To estimate the mean proportion of misreferrals in controls, we first tested whether there was a difference in the mean proportions of misreferrals between hands. If no significant difference was found, the data were averaged across hands. Comparison of controls against patients was done using an unpaired *t* test, unless either the data or the residuals of the original test were non-normally distributed. In this case, a Mann–Whitney test was used.

Second, we wanted to evaluate whether there was any structure to the frequency of occurrence of misreferrals across the hand. Do some digits make more misreferrals than others, for example, and if so, does this pattern differ in nerve injury? To our knowledge, these questions have not been addressed.

To evaluate this, we tested whether the distribution of observed misreferrals was different from the expected distribution if all digits had the same probability of misreferrals. In other words, if all digits were equally prone to misreferrals. Notably, since the thumb was probed 50% less than all other digits, the expected distribution is 11.11% for the thumb and 22.22% for digits D2-through-D5. Two chi-squared tests for goodness of fit were performed. One test was used to evaluate whether the distribution of misreferrals in healthy controls, averaged across hands, differed from the expected distribution. The second chi-squared test was used to evaluate whether the distribution of misreferrals for the injured hand of patients differed from the expected distribution (again, based on the null hypothesis that all digits are equally prone to misreferrals).

Finally, we also wanted to visualize directionality of misreferrals. To do so, we plotted the number of misreferrals that were made from a given digit according to the direction of the misreferral itself—i.e., which digit, or the palm, was the percept misreferred to? The data are expressed as the proportion of total misreferrals for the hand in question. This provides information on both the frequency of occurrence of misreferrals per digit and their directionality—i.e., where the perception of touch was mislocated. For healthy controls, the proportions were calculated separately per hand, and then the average was computed.

### Tests of Concurrent Validity

In the process of developing a new method, it can be useful to compare its outcome measures against those of established tests designed to assess similar constructs. Agreement across tests can strengthen confidence in the validity of the measures provided by the new method. We selected two methods for comparison, described next.

#### Sensory Rosen test.

The Rosen test is a standardized test of hand function after median/ulnar nerve repair, with established validity, reliability, and sensitivity (28, 29). The complete test includes separate sensory, motor, and pain domains. Only the sensory domain was tested here. Subtests include Semmes-Weinstein touch detection, two-point discrimination, shape-texture-identification (30), and the Sollerman hand function subtests 4, 8, and 10 (31).

We followed the procedures available at https://hakir.se/about-hakir/ (2018). Touch detection thresholds were taken using Semmes-Weinstein monofilaments, and two-point discrimination was taken using The Two-Point Discriminator (Exacta Precision & Performance, 2019 North Coast Medical, Inc.). Our shape-texture-identification and the Sollerman test materials were both produced in-house. The blinder box from the main experiment was used to block the participants’ view of their hand during the first three components of the sensory Rosen test (i.e., touch detection, two-point discrimination, and shape-texture-identification).

#### Modified Marsh method.

As discussed in the introduction, the modified Marsh method is a standardized test of touch localization after median/ulnar nerve repair. The test shows good sensitivity to impairment in nerve injury, with excellent test-retest and interrater reliability and external validity (18). Target locations in the main experiment of the current study were modeled after the zones of the modified Marsh method (Fig. 1*D*).

The current study followed standardized procedures (freely available on request at https://www.uea.ac.uk/about/school-of-health-sciences/research/resources-and-tools/the-locognosia-test). Participants were provided with a hand diagram (one each for the left and the right hand) that showed all 20 zones that could be stimulated (Fig. 1*D*). All zones were numbered and after stimulation, participants reported the number of the zone where they felt the stimulation.

Two trials were taken per zone and randomized for presentation. The number of zones tested depended on the type of injury. Median nerve patients were tested in zones 1 through 14. Ulnar nerve patients were tested in zones 15 through 20. Patients with both nerves injured were tested in all 20 zones. Both hands were tested. The zones tested for the uninjured hand were the homologous locations defined by the type of nerve injury, as described above. The injured hand was tested first.

Performance is measured as a score: 2 points for the correct zone; 1 point for an adjacent zone; 1 point for the homologous zone of a neighboring digit; 0 points for other responses. The Marsh score is then computed as the sum of points across trials. Since the zones tested differed for different patient types, see above, we converted raw Marsh scores to percentages based on the number of maximum points attainable per patient type.

On the day of testing, the modified Marsh method was completed last, and five patients could not complete this test due to time constraints. As noted above, patients underwent other tests on the same day, including functional MRI scans. We had run overtime with these four individuals.

#### Clinical questionnaires.

We also administered two additional standardized questionnaires: *1*) the Disabilities of the Arm, Shoulder, and Hand (DASH) outcome measure (32) and *2*) the short-form McGill Pain Questionnaire (SF-MPQ) (33).

The DASH questionnaire assesses the impact of upper-limb injury—not specific to nerve injury—on activities of daily living. The SF-MPQ assesses the type and level of pain caused by injury. Both questionnaires are patient-reported outcomes producing a single score.

## RESULTS

### Individual-Level Data Separates Injured from Healthy Hands

**Figure 2.**
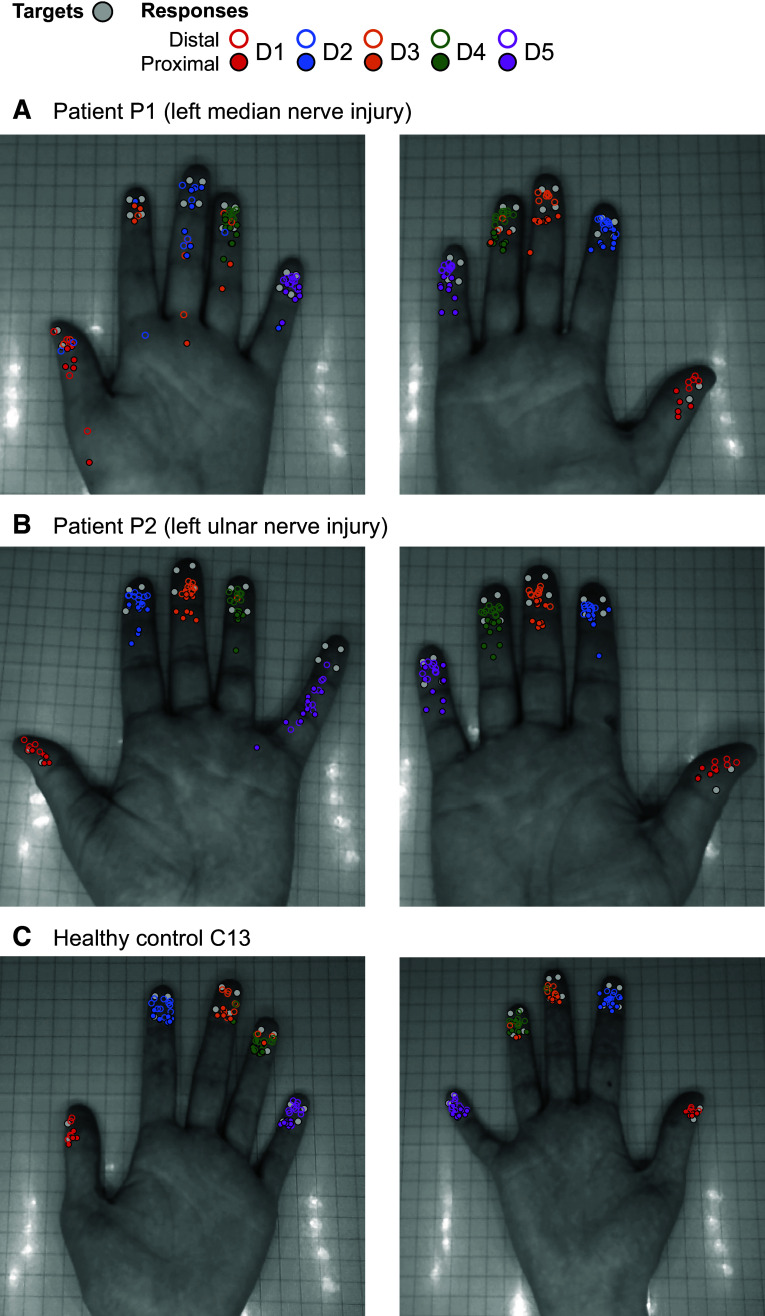
Individual-level data. Targets (gray circles) and responses (colored circles) of three sample participants overlaid on the photographs of their hand. The color of the responses indicates which digit the stimulation had been applied (see color key *inset*). *A*: this median nerve patient shows many misreferrals, especially from their injured D2 to D1 and D3. In addition, the responses show a large spread of error. There are also a few misreferrals from D3 to D4 on their uninjured hand. *B*: this ulnar nerve patient showed a large spread of responses specific to D5 of their injured hand. They make one misreferral to the palm and one misreferral from D3 to D4. *C*: healthy control participant. Note that even healthy controls can show some misreferrals, especially between D3 and D4 as can be seen in this example participant, for both hands.

Figure 2 provides a qualitative overview of the information provided by the digital photograph method, at the individual participant level. Data from two patients and one control participant are shown as examples. Responses are overlaid with targets in raw x- and y-coordinates, shown separately per hand. Different colored responses indicate which digit was stimulated.

A number of observations can be made. First, there is a greater spread of responses for the patient’s injured hand, consistent with an increased error of localization. Second, increased error of localization appears to be restricted to the projection territory of the injured nerve. *Patient P2* provides a clear example. In this patient, the ulnar nerve was injured and the spread of responses for *digit 5* is relatively pronounced (Fig. 2*B*). Localization performance for the uninjured hand of patients, and for either hand in controls, is comparatively better; responses tend to cluster close to targets. Third, and perhaps less obvious, error of localization appears to show a proximal bias; off-target responses are generally seen as more proximally located. This proximal shift in response error is apparent in patients and controls. Lastly, both patients and controls make misreferrals, yet some patients show higher numbers of misreferrals (Fig. 2*A*). These apparent differences are again specific to the injured hand. Below, we offer more detailed analyses of misreferrals (see results, *Misreferrals*, Fig. 5).

To summarize, our method provides information about error of localization, its potential systematic directionality, and misreferrals. This information separates injured from healthy hands at the individual participant level, as qualitative descriptive observations. Next, we investigate whether these observations hold quantitatively, at the group level.

### Group-Level Data Separates Injured from Healthy Hands

**Figure 3.**
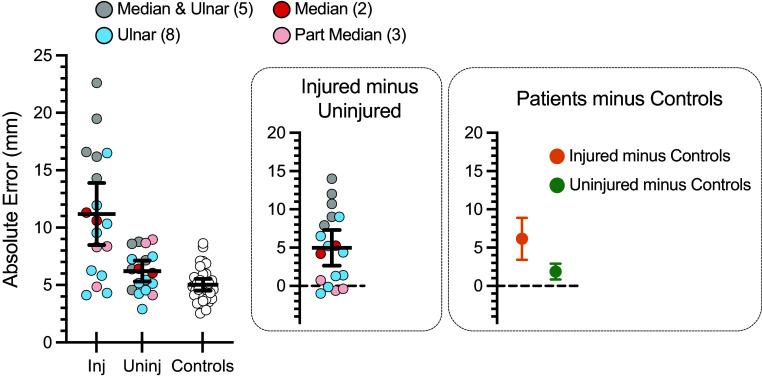
Group-level results: absolute error of localization. Mean values of absolute error of localization are shown for patients (*n* = 18) and controls (*n* = 33). Inj, injured territory; Uninj, homologous uninjured territory. Individual datapoints are mean estimates per participant and error bars are 95% confidence intervals around the group means. The same data are shown as difference scores, computed as the difference in absolute localization error between Inj minus Uninj sides (first *inset*). The second *inset* shows the mean differences between groups, with estimated 95% confidence intervals around the differences, respectively.

Figure 3 plots absolute error of localization. Patient data are shown for responses to targets within the injured nerve territory of the injured hand, “Inj,” and the homologous targets on the uninjured side, “Uninj.” These same data are also shown as difference scores—computed as the difference in absolute localization error between Injured minus Uninjured sides (first *inset* in Fig. 3). Positive difference scores indicate a greater error for the injured side, consistent with impairment due to injury.

The results reveal sensitivity to impairment. The error of localization is significantly increased for responses to targets within the injured nerve territory relative to the homologous targets on the patient’s uninjured side [*t*(17) = 4.5, *P* < 0.001, η_p_^2^ = 0.54]. In other words, as a group, the patients are significantly worse at localizing touch on their injured hand.

Visualizing these effects as difference scores also shows that not all patients are impaired. Negligible differences are observed for a subset of patients—all three patients with part-median-nerve repairs, and four patients with ulnar nerve repairs. As described below, an apparent absence of impairment in touch localization within a given individual tends to agree with other independent measures of functional recovery, namely, sensory Rosen scores. Those patients who show negligible differences in absolute error of localization between injured and uninjured sides also tend to show high levels of functional return according to their scores on the sensory Rosen test (see results, *Comparison with sensory Rosen scores*).

Figure 3 also plots the absolute error of localization in healthy controls. Controls’ data reflect responses to targets matched to those of the patient group based on the proportions of patients across subgroups defined by injured nerve (see methods, *Analyses* for details). Controls data are averaged across hands since no reliable differences between the dominant and nondominant hands are observed [*t*(32) = 1.1, *P* = 0.28, η_p_^2^ = 0.04].

These results further demonstrate the sensitivity of the digital photograph method to nerve impairment. Absolute error of localization for the injured hand of patients is significantly increased relative to that of healthy controls [Welch-corrected *t*(18.3) = 4.7, *P* < 0.001, η_p_^2^ = 0.55].

Unexpectedly, although much smaller in magnitude, we also find a statistically reliable difference in performance between the uninjured hand of patients and healthy controls [Welch-corrected *t*(29) = 2.4, *P* < 0.05, η_p_^2^ = 0.16]. Localization is worse for the uninjured hand of patients. There are several possible interpretations to consider with regard to these unexpected findings, including potential confounding factors.

First, although we include a control task designed to catch motor problems (see methods, *Control task*), it is possible that patients experienced difficulties operating the mouse with their injured hand and that our control task failed to capture this. This could happen, for example, if difficulties were to arise later in time, after the control task was completed. Even if only minor, these challenges could, in principle, negatively impact performance. Worse localization for the uninjured hand would arise. This could help explain our unexpected findings. This is a limitation of the digital photograph method. In the extreme, if controlling the mouse to record responses with the injured hand is too problematic (e.g., painful), then testing of the uninjured hand may not be possible.

Second, the current tests were carried out in the context of a broader study involving additional experiments, and not all controls underwent the same tests, or test schedule, as patients. Specifically, all patients underwent functional MRI testing before completing the behavioral experiments reported here, and all tests were performed on the same day. This was not the case for all controls. Four control participants did not complete fMRI testing, and 10 controls completed fMRI and behavioral tests on separate days. Perhaps, patients were generally more fatigued than controls at the time of completing the digital photograph method, and this could help to explain why touch localization performance with their uninjured hand was worse than controls.

Finally, although similar, the mean age and range of age of our patient group are larger than that of our control group. Perhaps, touch localization performance declines with age, and this could explain why, as a group, patients perform worse with their uninjured hand. Motivated by this possibility, we tested for evidence of a relationship between the mean absolute error of localization and age. No reliable relationship was detected [healthy controls and average hand performance, r(31) = 0.12, *P* = 0.50, r^2^ = 0.02; patients and uninjured hand performance, r(16) = 0.07, *P* = 0.78, r^2^ = 0.01; combined controls and patients, considering only the uninjured hand performance, r(29) = 0.17, *P* = 0.22, r^2^ = 0.03]. This suggests that age differences between groups are unlikely to explain why patients performed worse with their uninjured hand relative to controls.

Altogether, it remains unclear why the uninjured hand of patients performed worse than healthy controls. What is clear, however, is that these effects were minimal compared with the effects observed due to nerve injury. The injured hand of patients showed far worse performance compared with both their uninjured hand and with the healthy hands of controls. Clearly, these effects are due to nerve injury and cannot be explained by the potential confounding factors considered above.

### Digit-Specific Analysis Separates Patient Subgroups

To evaluate whether our method is sensitive enough to distinguish patient subgroups according to which nerve is injured, we performed a separate digit-specific analyses of error of localization. Focusing on *digit 2*, patients with median nerve repairs are defined as an “expected impaired” group, whereas patients with isolated ulnar nerve injuries are defined as a patient control group. The converse is true for our analyses of *digit 5*. Patients with ulnar nerve injuries comprise the “expected impaired” group, whereas those with isolated median nerve injuries comprise the patient control group. We also include analyses of data from healthy controls, similar to above.

**Figure 4.**
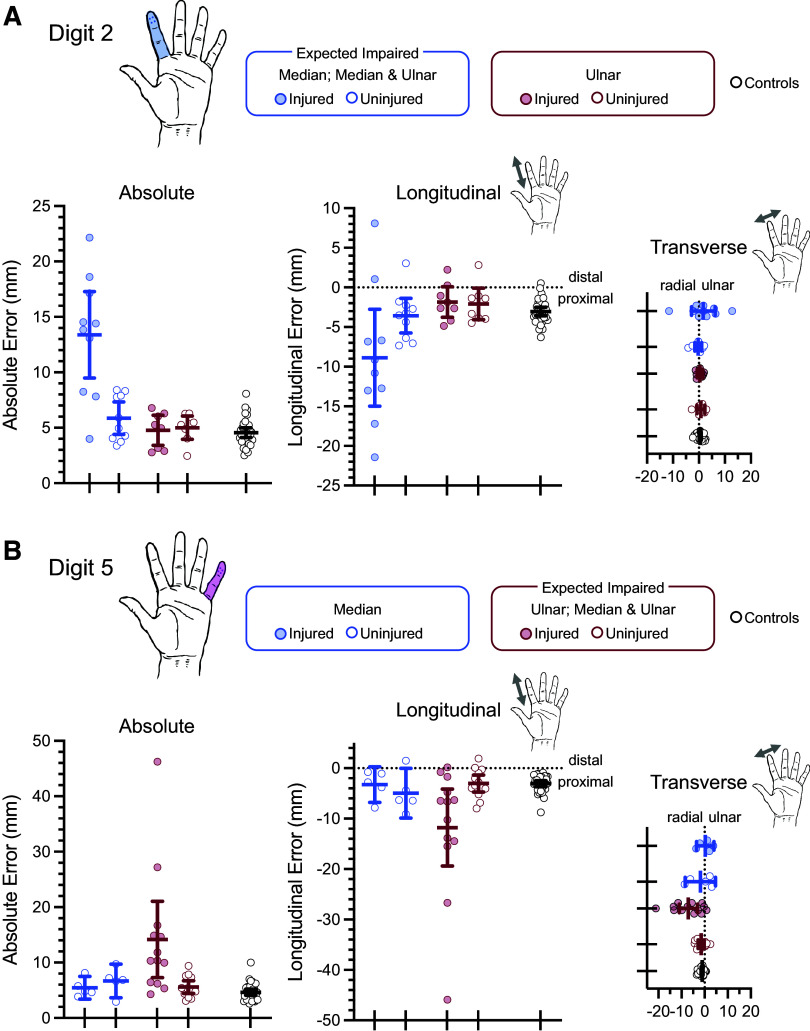
Digit-specific results: absolute and directional error of localization. The mean absolute error of localization and its longitudinal and transverse components are shown for patient subgroups and controls for *digit 2* (*A*) and *digit 5* (*B*). Patient subgroups are defined as “expected impaired” depending on the nerve injured and digit examined. *A*: data for *digit 2*. Patients with median nerve injuries are expected to show impairments (*n* = 10, expected impaired; *n* = 8, ulnar; *n* = 33, healthy controls). *B*: data for *digit 5*. Patients with ulnar nerve injuries are expected to show impairments (*n* = 13, expected impaired; *n* = 5, median; *n* = 33, healthy controls). Individual datapoints are mean estimates per participant and error bars are 95% confidence intervals around the group means.

The results reveal sensitivity to impairment at the digit-specific level, depending on which nerve is injured. Absolute error of localization for responses to stimulation of *digit 2* is increased for patients with median nerve injuries, but not for those with isolated ulnar nerve injuries (Fig. 4*A*). This is confirmed statistically as a significant difference between the injured versus uninjured hands in the “expected impaired” group [*t*(9) = 3.8, *P* < 0.005, η_p_^2^ = 0.67] and between the injured hand of the “expected impaired” group and healthy controls [Welch-corrected *t*(9.2) = 5.1, *P* < 0.001, η_p_^2^ = 0.74]. No reliable differences are observed in the patient control group, with isolated ulnar nerve injuries [injured vs. uninjured hands: *t*(7) = 0.60, *P* = 0.57, η_p_^2^ = 0.05; injured vs. healthy controls: Welch-corrected *t*(9.1) = 0.34, *P* = 0.74, η_p_^2^ = 0.01].

The complementary results are observed for analyses of *digit 5* (Fig. 4*B*). Here, impairment is seen for patients with ulnar nerve injuries [injured vs. uninjured hands: *t*(12) = 3.1, *P* < 0.01, η_p_^2^ = 0.44; injured vs. healthy controls: Welch-corrected *t*(12.2) = 3.0, *P* < 0.05, η_p_^2^ = 0.43], but not for those patients with isolated median nerve injuries [injured vs. uninjured hands: *t*(4) = 1.0, *P* = 0.37, η_p_^2^ = 0.20; injured vs. healthy controls: Welch-corrected *t*(5.1) = 1.0, *P* = 0.35, η_p_^2^ = 0.17]. Again, this demonstrates sensitivity to impairments according to which nerve is injured and which digit is examined. Localization impairments are identified for digits within the territory of the injured nerve; touch localization for digits outside the territory of the injured nerve appears normal.

As an additional step, we evaluate whether these results depend on including the patients with injuries to both nerves—the “median & ulnar” group. As these patients are included within the “expected impaired” group for both analyses, *digit 2* and *digit 5*, it is possible that the above results are driven entirely by this group. To evaluate this, we repeated analyses of *digit 5* using only isolated ulnar nerve patients as the “expected impaired” group. The results support our conclusions above; again, we find evidence for specificity. Performance is impaired for the injured versus uninjured sides [*t*(7) = 2.6, *P* < 0.05, η_p_^2^ = 0.49] and for injured versus healthy controls [Welch-corrected *t*(7.1) = 2.4, *P* < 0.05, η_p_^2^ = 0.45]. It was not possible to conduct a similar complementary analysis for *digit 2* given too few patients with isolated median nerve injuries. Nonetheless, we are confident from these findings that our new digital photograph method has the potential to identify impaired touch localization at the level of individual digit responses according to whether the ulnar or median nerve is impaired.

Figure 4, *A* and *B*, also shows the error of localization for *digits 2* and *5* as longitudinal and transverse components, respectively. These analyses were done to explore the added potential of our method. We wanted to test whether the digital photograph method could identify potential biases in touch localization responses—evidence for systematic directionality in the error of localization. Although prior reports suggest a proximal bias in localization error on the volar surface of the hand in healthy controls (15), we had no a priori predictions regarding a possible change in such biases resulting from nerve injury. These analyses were thus exploratory.

Several inferences can be made from these results. First, most of the localization error is expressed along the length of the digits. For both patients and controls, localization error is greater in the longitudinal relative to the transverse direction, and this is true for both *digits 2* and *5*. This is unsurprising given that there is more “room” to make errors along the length of the digit relative to its width. Second, in healthy controls, the error in the longitudinal direction shows a significant proximal bias [D2: *t*(32) = 11.7, *P* < 0.001, η_p_^2^ = 0.81; D5: *t*(32) = 10.3, *P* < 0.001, η_p_^2^ = 0.77]. This pattern is generally unchanged in the case of the injured hand of the “expected impaired” group, yet not all tests reach significance following corrections for multiple comparisons. Third, no evidence for a directional bias in localization error along the transverse component is observed in healthy controls for *digit 2* [*t*(32) = 2.3, *P* = 0.03, η_p_^2^ = 0.14], yet a very small (mean = −1.1 mm) but statistically reliable bias is observed in the radial direction for *digit 5* [*t*(32) = 6.3, *P* < 0.001, η_p_^2^ = 0.55]. For patients, this radial bias for *digit 5* is also observed and is more pronounced in magnitude (mean = −7.05 mm) due to injury of the ulnar nerve [*t*(12) = 3.9, *P* < 0.005, η_p_^2^ = 0.56]. This is of potential interest; yet, with no a priori expectation regarding this finding, we provide no further interpretations here. Future investigations are needed to replicate and further explore the potential significance of these results.

### Misreferrals

Overall, misreferrals were made infrequently. This makes quantitative statistical evaluations challenging. Nonetheless, we report the following results for completion and to demonstrate the potential of the digital photograph method to capture these details.

**Figure 5.**
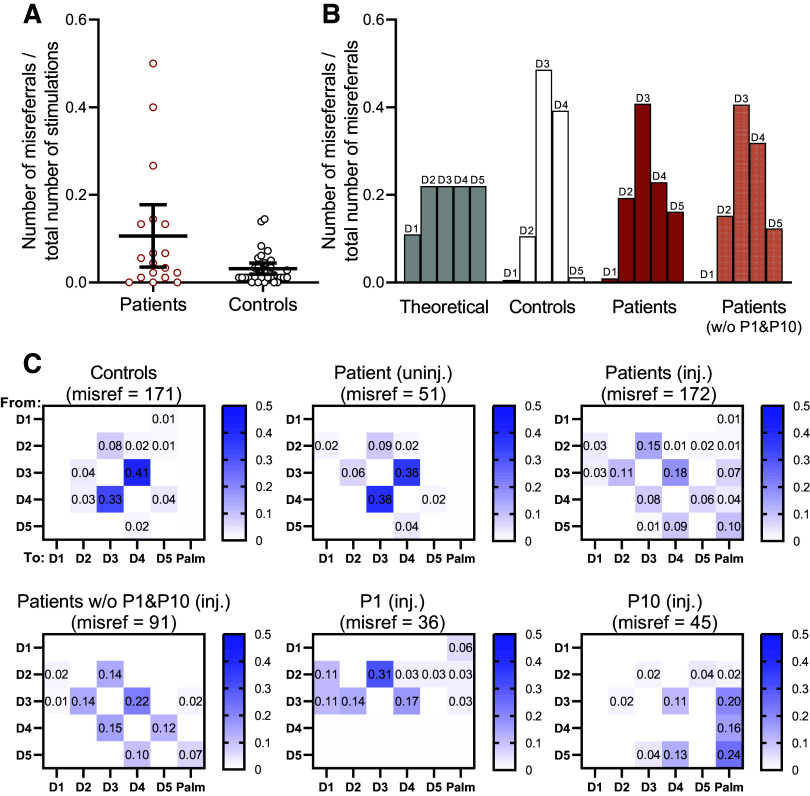
Misreferrals. *A*: number of misreferrals per participant expressed as the proportion of total stimulations. Patient data are from the injured hand. Group mean estimates are shown with 95% confidence intervals. Individual datapoints indicate individual participant data (*n* = 18, patients; *n* = 33, healthy controls). *B*: number of misreferrals per digit expressed as the proportion of the total number of misreferrals per group. The “theoretical” distribution is the expected distribution if each digit was equally likely to make misreferrals. Patient data are shown with and without *P1* and *P10* included. *C*: confusion matrices showing the number of misreferrals between segments of the hand expressed as the proportion of the total number of misreferrals. The vertical axes indicate where the misperception of touch was “referred from,” and the horizontal axes indicate where the misperception of touch was “referred to.” Patient data are shown for the uninjured and injured hand. For the injured hand, the data are shown with and without *P1* and *P10*, and the individual data for *P1* and *P10* are also shown.

Figure 5*A* shows the number of misreferrals expressed as the proportion of the total number of trials for the injured hand of patients and for the average between hands in controls. Statistical comparison of the group mean proportions reveals no reliable differences (Mann–Whitney *U* = 203, *P* = 0.19, η_p_^2^ = 0.22). The number of misreferrals is generally very low for both groups. Most controls make only one or two misreferrals or none, and only two control participants make misreferrals at a frequency ranging between 10% and 15%. Patients also generally make few misreferrals. However, three patients stand out from the rest of the patient group, and from controls, showing high numbers of misreferrals. *P8*, *P1*, and *P10* make misreferrals at a frequency of 26%, 40%, and 50%, respectively. Thus, tracking the frequency of misreferrals with the digital photograph method identifies individual patients showing strikingly high numbers of misreferrals, yet at the group level, no reliable differences between patients and controls are observed.

Despite the high interparticipant variability and generally low frequency of misreferrals in either group, we decided to follow through with our planned analyses to evaluate the structure of observed misreferrals across the hand. First, we test whether the observed distribution of misreferrals in either group differs from an expected—theoretical—distribution if all digits are equally likely to make misreferrals. Our findings indicate that the distribution of misreferrals in both patients and controls differs from the expected distribution [Fig. 5*B*; controls: χ^2^(4) = 137.1, *P* < 0.001, patients: χ^2^(4) = 60.22, *P* < 0.001, patients without *P1* and *P10*: χ^2^(4) = 51.4, *P* < 0.001]. In other words, not all digits are equally prone to misreferrals. In controls, the majority of misreferrals arise following stimulation of either D3 or D4. Patients also depart from the theoretical distribution, yet relative to controls, show more misreferrals for D2 and D5. Patient data are shown with and without *P1* and *P10* included, as these two individuals showed abnormally high numbers of misreferrals, as described above.

Finally, the directionality of misreferrals is visualized by plotting the number of misreferrals per digit according to the direction of the misreferral itself—i.e., which digit, or the palm, was the experience of touch mislocalized to. This generates a five-by-six matrix, where the five rows indicate “misreferred from” and the six columns indicate “misreferred to.” These data are shown separately for controls and for the injured and uninjured hand of patients, expressed as the proportion of total misreferrals per hand (Fig. 5*C*). Patient data are presented with and without *P1* and *P10*, and individual-level data for these two patients are provided.

Although purely qualitative, some observations can be made. In both healthy controls and for the uninjured hand of patients, the majority of misreferrals involve confusion between D3 and D4, accounting for 74% of all misreferrals. This suggests that D3 and D4 are disproportionately prone to confusion in the healthy hand. This pattern breaks down for the injured hand of patients. The frequency and directionality of misreferrals are more widely distributed and misreferrals are made to the palm. Examining these data separately for patients *P1* and *P10* is useful. Here, we see obvious departures from the pattern observed in controls and the uninjured hand of patients. Although merely descriptive, this highlights the added potential of the digital photograph method—individual patients showing high numbers and/or atypical patterns of misreferrals can be identified. This information could be of value, for example, to identify individual patients for further study and/or specialized treatment considerations.

### Tests of Concurrent Validity

Good practice in the development of a new method is to evaluate its outcome measures against those acquired from established tests thought to capture the same or similar constructs. Agreement across tests can strengthen confidence in the validity of the measures provided by the new method. The sensory Rosen test and the modified Marsh method were chosen for comparison. Both tests are known to evaluate meaningful hand function following ulnar/median nerve injury. As such, we expected the outcome measures of our new digital photograph method to relate to those of both tests.

To perform these comparisons, the outcome measure examined from the digital photograph method was taken as the mean error of localization expressed as the difference between injured and uninjured hands (see results, *Group-Level Data Separates Injured from Healthy Hands*; Fig. 3, first *inset*). Positive values reflect worse performance—greater localization error—for the injured side. In other words, these values stand as a measure of touch localization impairment. Values near zero suggest no impairment.

#### Comparison with sensory Rosen scores.

The sensory Rosen test produces a single composite score, with subtests that evaluate both low- and high-level functions (see methods, *Sensory Rosen test* for details). Higher sensory Rosen scores indicate better function. The test does not evaluate touch localization.

**Figure 6.**
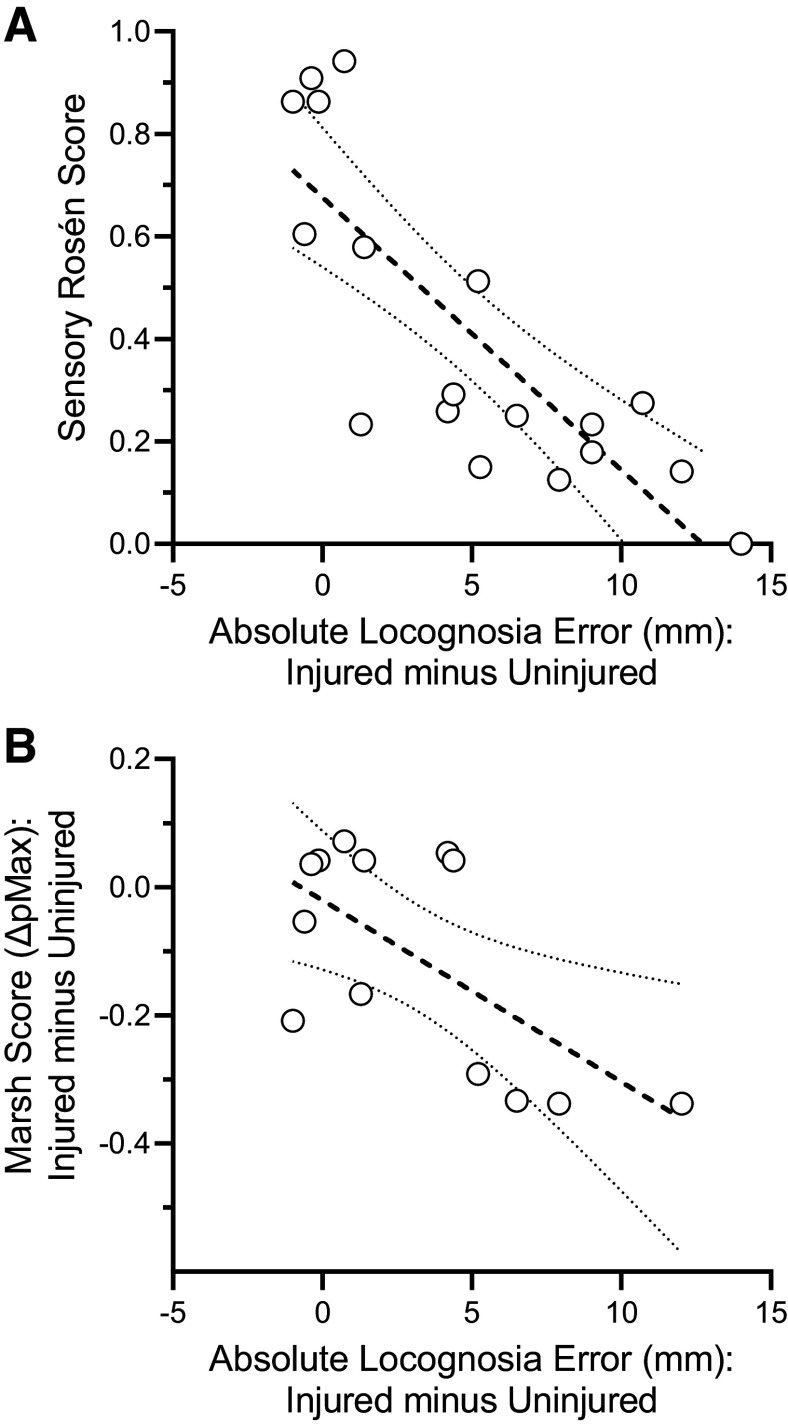
Tests of validity. *A*: comparison between sensory Rosen scores and localization error derived from the digital photograph method. Localization error is expressed as the difference between performance for the injured minus homologous uninjured territories (*n* = 18 patients). *B*: comparison between the Marsh scores and localization error derived from the digital photograph method. Both measures are expressed as the differences between performance for the injured minus homologous uninjured territories. Regression lines are shown with 95% confidence intervals (*n* = 13 patients).

Correlational tests reveal a strong significant relationship between our measures of touch localization from the digital photograph method and sensory Rosen scores [r(16) = −0.81, *P* < 0.001, r^2^ = 0.66] (Fig. 6*A*). The extent of impairment captured by each test is related. Patients with greater touch localization impairments also tend to perform poorly on the sensory Rosen test. This suggests that our new digital photograph method and the sensory Rosen test measure related constructs, sensitive to nerve injury.

#### Comparison with modified Marsh scores.

The modified Marsh method is a standardized test of touch localization, producing a single score per hand (see methods, *Modified Marsh method* for details). These scores are expressed as a percentage of best possible performance; values of 100 indicate no errors.

For comparison against the digital photograph method, modified Marsh scores were expressed as the difference between injured and uninjured hands. This makes the outcome measures from the two methods conceptually similar. In the case of the modified Marsh scores, negative difference values, calculated as injured minus uninjured scores, indicate greater impairment.

Our findings reveal a significant correlation between the two outcome measures [r(11) = −0.65, *P* = 0.016, r^2^ = 0.43]. Touch localization performance as evaluated by the digital photograph method relates to touch localization performance as evaluated by the modified Marsh method. This makes sense, and, overall, strengthens confidence in the validity of the measures derived from our new digital photograph method.

## DISCUSSION

We develop a new method for measuring touch localization and evaluate its value for use in nerve injury. Our method enables detailed quantification of the error of localization and its directional components, separate from misreferrals—errors made across digits, or from a digit to the palm. Our results show that nerve injury increases the error of localization and suggest that these impairments are restricted to the territory of the repaired nerve. A few patients also show abnormally high numbers of misreferrals, and the pattern of misreferrals in patients departs from that observed in healthy controls. We also find close agreement between our new measures of touch localization and the well-established, validated sensory Rosen scores, commonly used to evaluate hand function after nerve repair. We discuss the significance of our findings, as well as the value and future applications of the digital photograph method.

The characterization of touch localization in nerve injury is of key importance. The method we develop in the current study offers far more rigorous and detailed assessment than previous methods provide. Our method makes it possible to acquire multiple measurements from the same skin locations in a repeatable and efficient manner. The error of localization can be quantitatively examined as absolute and directional components, distinguished from misreferrals, which can also be examined for frequency of occurrence and directionality. This provides a rich profile of information at the level of individual participants and across different parts of the hand. Below, we argue that such rich detail is necessary to evaluate certain unanswered research questions of great significance and that the method we develop here is particularly well suited for this purpose. Our current findings also provide new and valuable fundamental insights. We discuss these contributions first.

### Fundamental Insights

Our findings make numerous new contributions to better understanding the functional consequences of nerve transection injuries. Despite the abundance of single-case observations highlighting abnormal touch localization in nerve injury and its recognized significance in tracking nerve regeneration (13), only one previous study provides quantitative data on the error of localization. Using the red-lens method, Braune and Schady (16) evaluate the error of localization in a group of 11 patients who underwent median/ulnar repairs. Surprisingly, they found normal localization at the distal pads of the digits, whereas increased error was found for the middle and proximal pads. The authors suggested that normal performance at the fingertips reflects compensation via central mechanisms, in alignment with their greater significance in active touch. Our results sharply contradict these findings and conclusions. We find that nerve injury leads to significant and lasting impairments in touch localization at the distal digit pads, challenging the notion that central-level mechanisms can fully compensate for such impairments.

Several inherent limitations of the red-lens method may help to explain these discrepancies. In the red-lens method, participants communicate their responses by touching their skin. With full vision available, they use a pen to mark where they felt they were touched by the experimenter. This provides an opportunity for participants to calibrate differences between the experience of touch caused by the stimulation event and that caused by their response, which is also accompanied by vision. Furthermore, in the red-lens method, the marks made by participants are visible and remain visible throughout the test. These features may improve localization accuracy, and, in the context of evaluating patients with nerve injuries, even obscure impairments.

With the digital photograph method, these concerns do not apply. Participant responses are made on an image of their hand, so no opportunity for calibration via feedback is available. And, once a given response is made, its location is no longer visible, ensuring that the visibility of prior responses does not influence current responses.

With the red-lens method, it is also inherently difficult to repeat measurements from the same skin locations. In the study by Braune and Schady (16), the estimates of localization error at the distal digit pads are derived from a single trial. This raises concerns regarding their reliability. In contrast, the digital photograph method enables efficient resampling of measurements at the same locations. This is a major strength. In the current study, numerous repeated measurements were taken to estimate the error of localization for each digit. As such, compared with Braune and Schady (16), our findings are based on significantly more rigorous evaluations.

With these considerations in mind, we are confident about our current findings and conclusions. Nerve injury results in significant impairments in the ability to localize touch at the distal digit pads. Notably, this result is also consistent with findings using an “area of localization” method known as the modified Marsh method (18). Although not measuring the error of localization, this method also targets the distal digit pads, and results from several independent studies reveal impaired touch localization following median/ulnar nerve repairs (18, 20–22). Taken together, these and the current results firmly challenge the idea that central-level mechanisms can fully correct for such impairments.

Our findings also provide new insights as to the specificity of localization impairments in nerve injury. Impairment is restricted to digits within the territory of the injured nerve. Localization is normal for digits outside the injured territory. To our knowledge, this is the first empirical demonstration of this level of specificity, validating prior conclusions drawn from qualitative observations in single patient cases (13). This finding also demonstrates the sensitivity of our new method. It seems likely that future studies could use the digital photograph method to evaluate localization impairments in less severe conditions, such as digital-nerve cuts and chronic nerve compression.

Our findings also reveal evidence of a radial bias in the directionality of localization errors following ulnar nerve injuries. As this bias was not predicted, we are hesitant to provide further interpretation of its potential significance. Nonetheless, this result highlights the additional capacity of the method—it is possible to investigate predictions regarding systematic directionality of localization errors. By way of example, we are aware of findings from brain injury cases showing systematic biases in touch localization (34, 35). Our digital photograph method could be used to further study these phenomena.

Finally, we also provide new support for the argument that touch localization is functional. We find a strong significant correlation between the error of localization and sensory Rosen scores: better touch localization is associated with better performance on the sensory Rosen test. The sensory Rosen test includes measures of high-level manual function and fine movement dexterity, as well as haptic object shape and texture recognition. These results reinforce previous findings and conclusions: touch localization in nerve injury is a good predictor of performance on high-level functional tests such as the Moberg pickup test (19) and activities of daily living (20). Taken together, touch localization is a valid index of meaningful function in nerve injury and should be considered important for clinical assessment and evaluation of treatment efficacy. In developing our new digital photograph method, we provide a more comprehensive and rigorous means for evaluating touch localization than previously available.

### Applications

One of the driving motivations for the current study was to develop a method that could be applied to the study of reinnervation errors. After a nerve has been cut and surgically repaired, regenerating fibers migrate out to the periphery without topographical guidance (4–7). New connections with end receptors are established at different locations relative to the preinjury organization. These rewiring events are known as reinnervation errors and are thought to significantly limit functional recovery. Despite their considered significance, however, the current understanding of reinnervations errors is remarkably limited. Part of the problem is that they are difficult to study.

Microneurography enables direct recording from peripheral nerves in humans, and although impractical for widespread use, may be the most definitive method available for the study of reinnervation errors. Recording from multiple afferents proximal to the site of repair in patients with median nerve injuries, Hallin et al. (36) document discontinuous sites on the hand where cutaneous stimulation evokes responses. In other words, regenerated afferent nerve fibers were found to exhibit multiple discontinuous receptive fields, in sharp contrast to the unitary receptive fields that characterize healthy nerves. These same patients showed large and numerous touch localization errors, including misreferrals. Both discontinuous receptive fields and distorted touch localization were interpreted as evidence of reinnervation errors.

The digital photograph method could help to significantly advance this line of research, combining touch localization with microneurography to study nerve injury. Although compelling, the results of Hallin et al. (36) lack quantitative validation. The neural receptive field properties and touch localization measures were not directly compared. Application of the digital photograph method in this context would not only enable detailed quantification of touch localization but also provide a platform for which neural receptive field properties could be defined in comparable units. Neural receptive field properties could be documented in the same “picture space” as touch localization data, allowing for comparisons between them. Agreement between measures would help to validate touch localization as a method for studying reinnervation errors. The digital photograph method enables the rich profiling of touch localization and means to exchange information between the experimenter and participant suitable to significantly advance this area of research.

The digital photograph method is also well suited to address questions regarding central-level changes following nerve injury. The primary somatosensory cortex is organized such that individual digits of the hand are represented separately, in a spatially ordered fashion (37, 38). This topographical organization is found to change in nonhuman primates after median nerve transection and repair (39–42). These changes in cortical topography are thought to reflect changes in the periphery and may relate to aberrant touch localization and reinnervation errors (41). To evaluate whether brain changes relate to abnormal touch localization in nerve injury, rigorous and detailed characterization of touch localization is essential. The digital photograph method enables this level of detail, surpassing the capabilities of previous methods.

Finally, given how well touch localization stands as a meaningful index of functional recovery following nerve injury, and its putative links to the quality of nerve regeneration, the digital photograph method provides a valuable new tool for evaluating patient recovery and treatment efficacy. Less rigorous and comprehensive methods may provide an incomplete or even misleading assessment.

### Limitations

The digital photograph method has several limitations. The method requires that two photographs of the participant’s hand be taken. The hand must be held flat against the base of the apparatus for these photographs, with the palm facing up and the fingers fully opened. Sometimes patients with nerve injuries have difficulties opening their hands. They may have a degree of flexion contracture related to associated tendon injuries and be unable to open their hand fully. The fingers tend to curl inward, toward the palm. Indeed, not included in the current paper, we have worked with two such patients unable to open their hand sufficiently to complete the digital photograph method. This suggests that a significant proportion of patients with nerve transection injuries may be unable to perform the digital photograph method. A useful future modification would be to validate different ways of completing the test from different hand postures. Also, although a more relaxed hand posture is assumed during testing, some degree of finger opening is necessary when targeting the locations tested in the current study (i.e., the distal digit pads). Otherwise, directing the monofilament with precision and at the appropriate angle for stimulation can be challenging.

With the current method, only the radial side of the thumb could be tested. The ulnar side of the thumb could not be made visible for the photographs, given the hand posture required. This represents a second limitation and further motivation to explore the viability of implementing different hand configurations in future modifications of the test. Perhaps multiple photos could be taken and used for recording responses, or different hand configurations could be used for different needs and purposes.

Third, in the current study, the time between successive photographs was 1 s. This means that the experimenter must remain vigilant; if the participant’s hand moves between photographs, the images must be retaken. This, of course, also requires that participants can keep their hand still while the photographs are taken. Certain clinical populations may find this challenging. Shortening the time delay between successive photographs would be a useful modification.

Finally, our current method is likely impractical for use in busy clinics. Procedures require too much time and involve specialized equipment. Forward steps include validating effects with fewer trials, automating target registration and data analyses, and improving overall ease-of-use, and the portability and affordability of materials.

### Concluding Remarks

Our findings significantly enhance our understanding of the functional consequences of nerve injury, providing a far more rigorous and intricate account of touch localization deficits due to median/ulnar nerve injuries than previously available. Our new method surpasses the capabilities of previous methods and provides a solid platform from which future investigations can build.

## DATA AVAILABILITY

Source data are openly available at: https://doi.org/10.17605/OSF.IO/QBRYZ.

## GRANTS

This work was supported by a Wellcome Trust Seed Award in Science (215186/Z/19/Z) to K.F.V.

## DISCLOSURES

No conflicts of interest, financial or otherwise, are declared by the authors.

## AUTHOR CONTRIBUTIONS

V.L. and K.F.V. conceived and designed research; M.W. and R.T. performed experiments; M.W., R.T., and K.F.V. analyzed data; M.W., A.M., F.M., S.W., E.J., V.L., and K.F.V. interpreted results of experiments; M.W., R.T., and K.F.V. prepared figures; M.W. and K.F.V. drafted manuscript; M.W., A.M., F.M., S.W., E.J., V.L., and K.F.V. edited and revised manuscript; M.W., A.M., R.T., F.M., S.W., O.O., L.B., E.J., V.L., and K.F.V. approved final version of manuscript.
